# Mechanophotonics: precise selection, assembly and disassembly of polymer optical microcavities *via* mechanical manipulation for spectral engineering†

**DOI:** 10.1039/d0na00560f

**Published:** 2020-10-14

**Authors:** Mari Annadhasan, Avulu Vinod Kumar, Dasari Venkatakrishnarao, Evgeniy A. Mamonov, Rajadurai Chandrasekar

**Affiliations:** School of Chemistry, University of Hyderabad Prof. C. R. Rao Road, Gachibowli Hyderabad 500046 India r.chandrasekar@uohyd.ac.in; Division of Quantum Electronics, Department of Physics, M. V. Lomonosov Moscow State University ul. Leninskiye Gory, 1 Moscow 119991 Russia

## Abstract

The advancement of nanoscience and technology relies on the development and utility of innovative techniques. Precise manipulation of photonic microcavities is one of the fundamental challenges in nanophotonics. This challenge impedes the construction of optoelectronic and photonic microcircuits. As a proof-of-principle, we demonstrate here that an atomic force microscopy cantilever and confocal microscopy can be used together to mechanically micromanipulate polymer-based whispering gallery mode microcavities or microresonators into well-ordered geometries. The micromanipulation technique efficiently assembles or disassembles resonators and also produces well-ordered dimer, trimer, tetramer, and pentamer assemblies of resonators in linear and bent geometries. Interestingly, an intricate L-shaped coupled-resonator optical waveguide (CROW) comprising a pentamer assembly effectively transduces light through a 90° bend angle. The presented new research direction, which combines mechanical manipulation and nanophotonics, is also expected to open up a plethora of opportunities in nano and microstructure-based research areas including nanoelectronics and nanobiology.

## Abstract 2

A combination of atomic force and confocal microscopy techniques seamlessly assembles and disassembles polymer optical microcavities.

## Graphical abstract

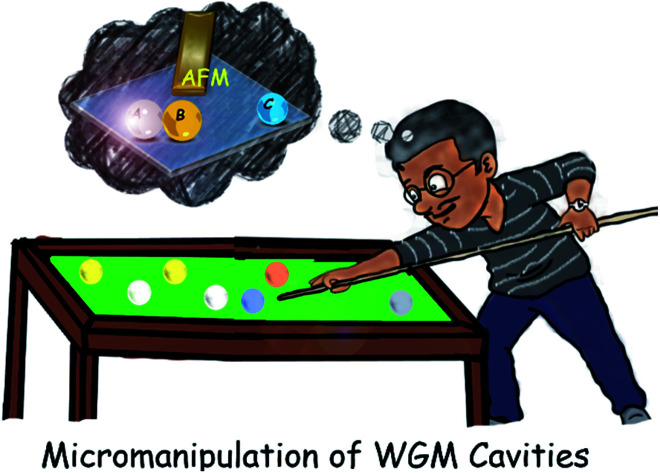

## Introduction

Mastering the technique of micromanipulation is crucial for the advancement of nanoelectronics, nanophotonics and nanobiology.^1^ In nanophotonics,^2–12^ micromanipulation is essential for the construction of all-optical circuits, optical sensors, laser-arrays, optomechanical devices, and so on.^1*h*,1*j*,4–7^ One of the major impediments towards the realization of such advanced nanophotonic devices is the lack of a suitable technique for accurate manipulation and precise integration of microcavities (a device which can trap light by resonant recirculation)^3^ to desired locations and various geometries for them to discharge complex optical functions. As a result, mostly, the function of merely a single microcavity has been demonstrated. Magnetic tweezers are only useful for microparticles with strong magnetic properties, and the precision of the method, however, is restricted by the magnetic hysteresis of the material.^1*f*^ Optical-tweezers are potentially expensive; therefore, direct use of an AFM cantilever tip for micromanipulation is still a promising, yet widely unexplored topic. Pierini *et al.* used atomic force microscopy (AFM) combined with optical-tweezers^1*g*^ to attach polystyrene microparticles to the cantilever for nanomechanical property studies. Shi *et al.* accurately positioned metallic and dielectric nanoparticles (NPs) using an AFM cantilever tip to construct optical nanocircuits.^1*h*^ Using the same technique, Shafiei *et al.* assembled metallic NPs to create nanosensors^1*i*^ and metamolecules.^1*j*^ NPs can also be dynamically manipulated on solid surfaces by pushing the particle with an AFM tip. To reduce the friction at the particle-surface (solid–solid) interface, which arises due to high van der Waals interaction, a surfactant layer was introduced between the particle and the surface.^1*k*^ Optical heating of the particle reduced the friction between the particle and the surface due to phase transition of the surfactants.

**Scheme 1:**
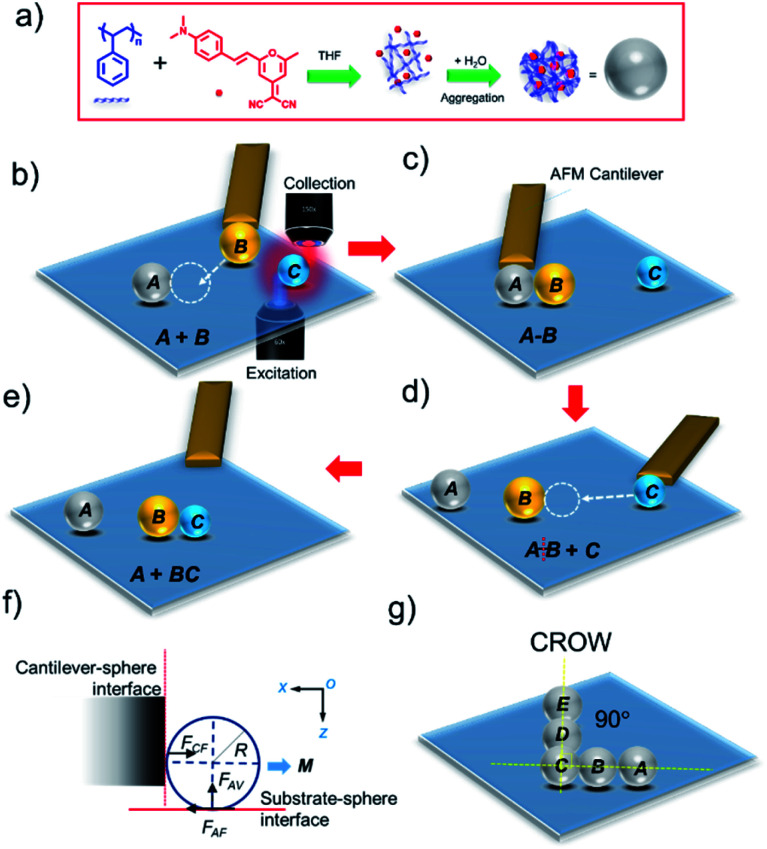
(a) Preparation of DCM-doped polystyrene optical microcavities *via* self-assembly. Schematic representation of the photonic molecule reaction: (b) AFM manipulation of cavity *B* towards cavity *A* using a tip-less cantilever. (c) Formation of an *A*–*B* dimer. (d) Separation of *A*–*B* and manipulation of cavity *C* towards cavity *B*. (e) Reaction of *C* with *B* and formation of the *B–C* photon molecule. (f) Interaction between the cantilever and microparticle during manipulation. *F*_CF_, *F*_AF_ and *F*_AV_ are the cantilever force, friction force, and normal force at the adhesive interface. *M* is the moment. (g) A coupled-resonator optical waveguide (CROW) prepared *via* the micromanipulation technique.

Though the electrostatic field involved between the microparticles and AFM cantilever tip is adequate to mechanically lift or push the particles, tip breakage and a small field of view limit this technique. Therefore, there is a demand for convenient and straightforward manipulation techniques to perform complex micromanipulation operations covering a larger area of the substrate using a tip-less cantilever. To carry out such manipulations of a smaller microparticle (diameter ∼3 μm) over a larger field of view, confocal optical microscopy (CFM) combined with AFM is required. We envisioned a combined CFM-AFM technique to monitor the movements (*x*, *y* and *z* coordinates) of the cantilever while *pushing* the microcavities to chosen trajectories by simultaneous viewing of both the cantilever and microcavities (Scheme 1b). Here, the movement of the particle is possible when the cantilever force is greater than the friction and normal force acting between the microparticle and surface interface (Scheme 1f). Additionally, an often discarded *tip-less cantilever* can also be effectively used as a mechanical manipulation tool to relocate a microparticle *via* sliding/or rolling from one site to another by pushing it.

One of the direct nanophotonic applications of the CFM-AFM technique is the fabrication of coupled microcavities by relocating, assembling and disassembling them mechanically. For this, self-assembled organic or polymer whispering-gallery-mode (WGM) microcavities^3,8^ are attractive candidates owing to their excellent mechanical stability.^3^ Two coupled microcavities are also termed as photonic molecule (PM)^9^ due to the formation of bonding and antibonding optical modes, akin to electronically hybridized orbitals formed between two bound atoms. PMs are attractive because depending upon the size of the individual cavities, the optical modes can be either enhanced or suppressed, or new modes can be formed. This size-dependency of PMs facilitates the use of PMs in frequency tunable lasers, optical filters, delay lines, and sensors and in future quantum computation technology. Ishii *et al.* fabricated GaInAsP based two size-mismatched twin micro-disk PMs to achieve bistable WGM lasing.^10^ Siegle *et al.* lithographically built a size-mismatched PM structure with tunable inter-cavity coupling gaps from dye-doped poly(methylmethacrylate) and excited the WGM lasing modes (so-called supermodes).^11^ However, until now, all the known PM structures have possessed a fixed geometry irrespective of the material used. As a result, it is not possible to perform *photonic molecule reactions* (PMRs), that is, assembly and disassembly of PMs with different cavities akin to bond making and breaking events observed in chemical reactions involving different atoms. Performing PMRs using differently sized optical cavities will bring new insights into the nascent field of PMs as these reactions will facilitate a fundamental understanding of the cavity size-effect on the appearance and disappearance of optical modes, new modes, and mode polarization.

Further, the CFM-AFM micromanipulation technique can also be extended to realize a sequence of microcavities, also known as a coupled-resonator optical waveguide (CROW) – a device theoretically proposed by Yariv *et al.*^12^ using WGM pentameric cavities. In contrast to other waveguides, in the CROW, light is transmitted from one cavity to another *via* evanescent coupling (EC) allowing a substantial reduction of the light speed and filtering of the optical modes depending upon the *Q*-factor and size of the cavities, respectively. An L-shaped CROW comprising pentameric cavities with a 90° bend angle between two arms can be used to transmit light through the right angle bend.

In this original work, we demonstrate the capability of the CFM-AFM-based micromanipulation technique in nanophotonic device applications. We use mechanically stable microspherical WGM cavities composed of red-emitting 4-(dicyanomethylene)-2-methyl-6-(4-dimethylaminostyryl)-4H-pyran (DCM) dye-doped polystyrene (PS) to carry out PMRs using three different microcavities of varying sizes. We precisely assemble *A* and *B* cavities into an *A*–*B* dimer and later disassemble them and assemble cavity *C* with *B* to create a new *B*–*C* dimeric cavity for spectral engineering. These reconfigurable dimeric optical cavities, due to their different sizes and the strong optical coupling between them, reveal frequency-shifted WGMs and a variation in the number of out coupled modes, mode splitting and polarization. In the end, we also show a sophisticated L-shaped CROW geometry that consists of five WGM cavities fabricated using mechanical manipulation transducing an optical signal at 90° bend angle.

## Experimental section

### Preparation of photonic microspheres

PS (10 mg) was dissolved in 4.0 mL of THF (HPLC grade) and sonicated for 5 min to dissolve the PS beads completely. To this 1 mg of DCM dye was added and mixed thoroughly by sonication for 5 min. Further, to this mixture, 1 mL of deionized water was added rapidly and left undisturbed for about 10 min to ensure the formation of microspheres. After this, 100 μL of this mixture was drop-cast on a clean glass coverslip, and the solvent was allowed to evaporate at rt to obtain DCM-doped PS microspheres (Scheme 1a).

### Confocal microspectroscopy

Microspectroscopy experiments were performed on a transmission mode set-up of a Wi-Tec alpha 300 AR laser confocal optical microscope and AFM microscope. Using a 300 groove per mm grating BLZ = 750 nm, the accumulation time was 5 s and integration time was typically 1.0 s. Ten accumulations were averaged for a single spectrum. A diode 405 nm laser (0.01 mW) was used as an optical excitation source. A 20× objective was used for recording the images, 60× for optically exciting the sample and 150× for collecting the photoluminescence (PL). All experiments were performed under ambient conditions.

### Micromanipulation of the polymer microparticles

Micromanipulation experiments were carried out using AFM combined with the CFM described above. The microparticles of choice were selected for the mechanical manipulation using a tip-less AFM cantilever (TipsNano: NSG10, Force constant 3.1–37.6 N m^−1^). Initially, the AFM cantilever was attached to the holder and aligned to the centre by using *x*/*y*/*z* direction control under a CFM. Then the cantilever was moved in the −/+*z* direction to reach the microparticles.

## Results and discussion

**Fig. 1:**
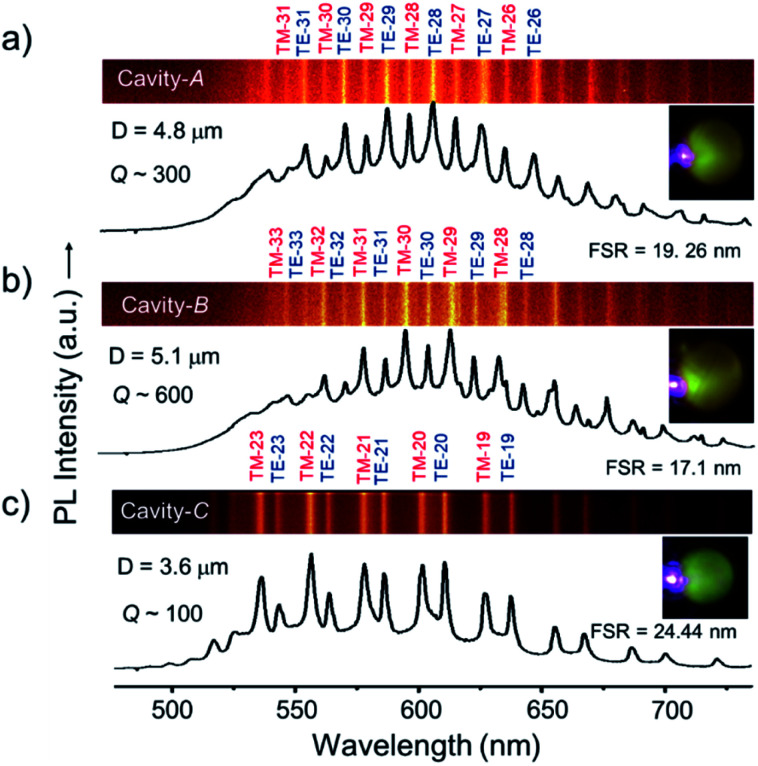
(a–c) PL spectra of *A*, *B*, and *C* microcavities displaying WGMs. The line spectra are shown in the top panels of each spectrum with calculated azimuthal mode numbers. The right insets show the optical microscopy image of the excited (*λ*_ex_ = 405 nm) cavities.

To perform PMRs, initially, we identified three microspheres, namely, *A*, *B* and *C* with estimated diameters (*D*) of 4.8, 5.1, and 3.6 μm, respectively, using the CFM. For the optical studies of microspheres, we used a continuous wave (CW) 405 nm laser in a transmission mode (bottom excitation and top collection with 60× and 150× objectives, respectively) geometry (Scheme 1b). Excitation of the edge of microsphere *A* produced red PL corresponding to the PL of DCM dye (Fig. 1a, right inset). The PL collected from the microsphere showed a series of pairs of sharp peaks, so-called WGMs, which arise due to multiple optical interferences of trapped light within the microspheres (Fig. 1a).^3^ We also presented the top view image of the spectrum, where each mode appears as a line in the PL spectrum. Finite differential time-domain (FDTD) simulations performed using Lumerical FDTD solutions software pointed out the presence of transverse electric (TE) and transverse magnetic (TM) polarized modes which arise as a result of the removal of the degeneracy.^3*a*,*d*^ The selected azimuthal TE/TM modes (radial mode number, *r* = 1) from TE-26 to TM-31 (the numbers correspond to polar *l* and azimuthal *m* mode numbers, *l* = *m*) obtained from the FDTD calculations are shown in Fig. 1a. The free spectral range (*FSR*) values of cavities *A*–*C* increased with respect to the decrease of the cavity size as per the relationship, *FSR* ∼ 1/*D*. The calculated *Q* factor [*Q* = *λ*/Δ*λ*; where Δ*λ* is the line width of a peak at full-width-at-half-maximum (FWHM) and *λ* is the wavelength of the peak] of cavities *A*–*C* exhibited a decreasing trend with values 300, 600 and 100, respectively, as per the *Q* ∼ 1/*D* relationship. Notably, the *Q* values of the TM modes were found to be smaller than those of the TE modes as a result of the larger FWHM of the latter type of mode. Similar experiments performed on a slightly larger sphere, *B*, revealed the WGMs in the PL spectrum with the TE/TM modes from TE-25 to TM-33. The much smaller microsphere, *C*, revealed WGMs with a clear set of TE/TM modes, which are identified as TE-17 to TM-23 with a much broader free spectral range in comparison to *A* and *B*.

**Fig. 2:**
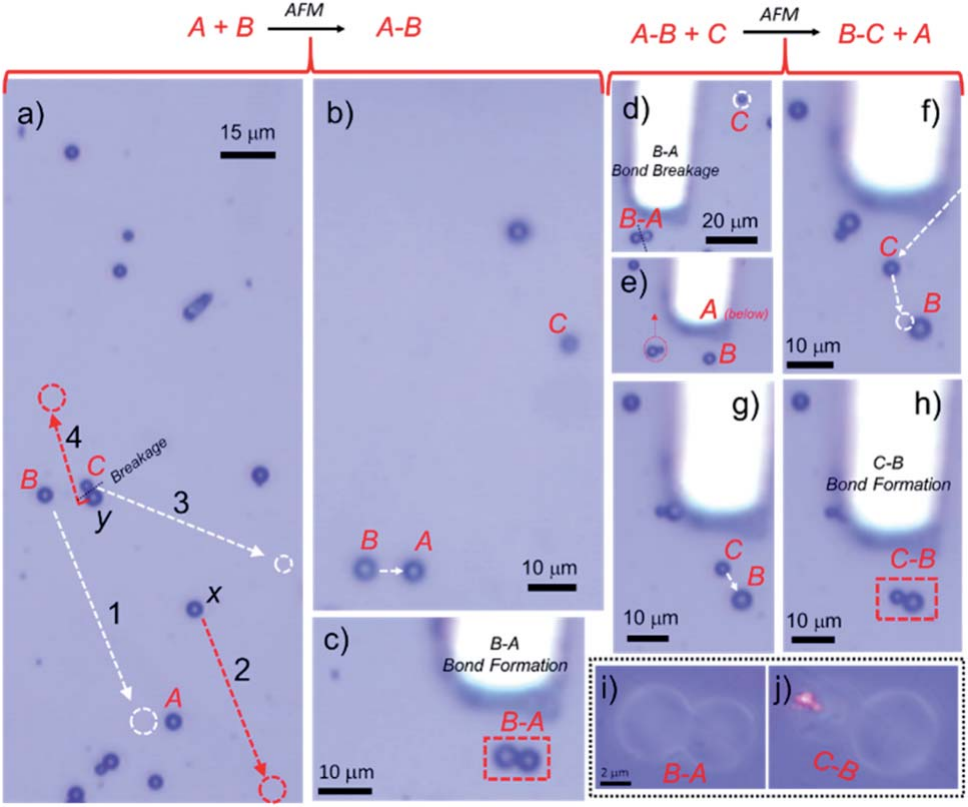
(a) AFM cantilever-assisted mechanical manipulation tracks of cavities *B* and *C* shown in white colour. The red arrows and circle show clearing of the unwanted cavities from the reaction center to other sites, respectively. (b) Micromanipulation of cavity *B* towards cavity *A* with an AFM cantilever. (c) Formation of an *A*–*B* dimer or photon molecule. (d–g) Breakage of *A*–*B* and manipulation of cavity *C* towards isolated *B*. (h) Reaction of *C* with *B* and formation of *B–C* photon molecules. (i and j) A close-up view of photon molecule *A*–*B* and *B–C*.

After confirming the photonic characteristics of individual cavities, to create an *A*–*B* PM, we identified cavity *B* located about 50 μm away from the physical reaction site (location of *A*) using CFM. Later, with an AFM (tip less) cantilever, cavity *B* was mechanically pushed and manoeuvred towards *A* (see label-1 in Fig. 2a). After careful positioning of *B* close to *A* (Fig. 2b), the former cavity was pushed near *A* to form a final contact (analogous to chemical bond formation between the two atoms), thereby forming an *A*–*B* or *B*–*A* molecule (Fig. 2c and i).

**Fig. 3:**
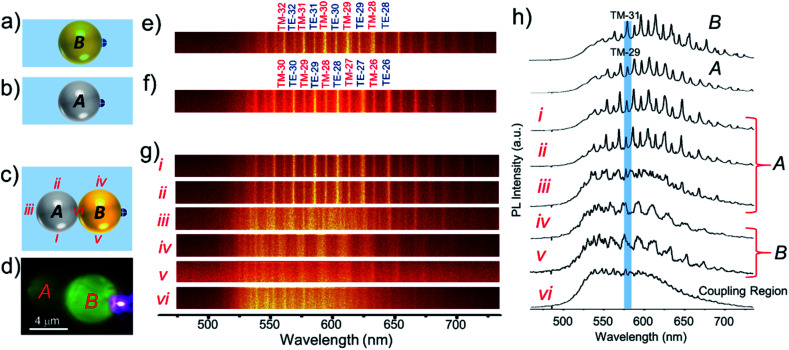
(a–c) Schematic representation of the top-views of microcavities *A* and *B* and the PM *A*–*B*, respectively. (d) Confocal PL microscopy image of photon molecule *A*–*B* with selective excitation of *B*. (e–g) WGM optical emission line spectra of cavities *A* and *B* along with azimuthal WGM numbers and photon molecule *A*–*B* collected at locations *i*–*vi*. (h) PL spectra of cavities *A*, *B* and *A*–*B* displaying WGMs. The labels *i*–*vi* show the spectra recorded at the different locations shown in (c).

The optical coupling between *A*–*B* cavities *via* an evanescent field was studied by optically exciting the extreme right edge of cavity *B* (Fig. 3c and d). The PL spectra at different positions of *A*–*B* cavities were collected at various locations labelled *i*–*vi* (Fig. 3c). The WGMs in the PL spectra of dimer *A*–*B* are shown in Fig. 3g and h (*i*–*vi*). In comparison to individual cavities, *A* and *B*, the *A*–*B* dimer revealed different WGMs. The modes recorded at positions *i* and *ii* were identical to the WGMs of cavity *A*. However, at location *iii*, which is the extreme opposite end of the excitation point, a completely different WGM spectrum from the spectrum of *A* was detected. This result confirms the optical communication between *A* and *B* cavities *via* evanescent field coupling (Fig. S2†). The spectra recorded at positions *iv* and *v* resembled each other very much, with modes of relatively larger linewidths; however, their spectral pattern was entirely altered compared to that of cavity *B*. At point *vi*, which is the coupling region of cavities *A* and *B*, the spectrum was nearly comparable to the one recorded at point *v* with broadened modes and mode shifts. The coupling between cavities in PMs can also translate to mode splitting depending upon the cavity *Q* factor. In the case of *A*–*B* dimers, at positions *iii*–*vi*, the modes with the azimuthal numbers 29 and 31 correspond to *A* and *B* broadened with the splitting. FDTD calculations supported the mode splitting by showing bonding and anti-bonding of modes from *A* and *B* with azimuthal numbers 29 and 31, respectively (Fig. S1†).

**Fig. 4:**
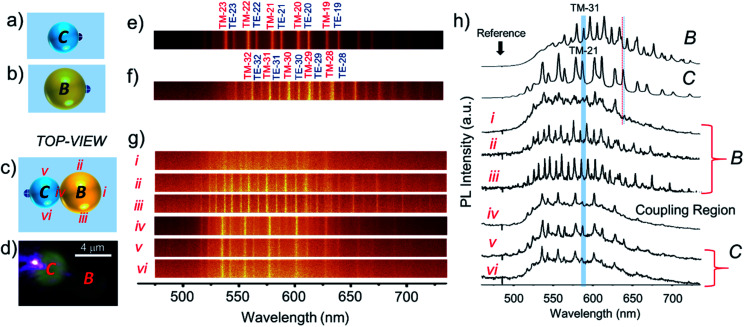
(a–c) Schematic representation of the top-views of microcavities *B* and *C* and the PM *B*–*C*, respectively. (d) Confocal PL microscopy image of the photon molecule *B–C* with selective excitation of *B*. (e–g) WGM optical emission line spectra of cavities *C* and *B* along with azimuthal WGM numbers and photon molecule *B–C* collected at locations *i*–*vi*. (h) PL spectra of cavities *C*, *B* and *B–C* displaying WGMs. The labels *i*–*vi* show the spectra recorded at the different locations shown in (c).

To carry out another PM reaction between cavity dimer *A*–*B* and cavity *C*, akin to a chemical reaction, *A*–*B* + *C* → *B*–*C* + *A*, first, it was essential to separate *A* and *B* from *A-B via* disassembly (bond breaking reaction). Secondly, a new cavity *C* needs to be connected to *B* to construct the *B*–*C* PM (bond forming reaction). To accomplish the PM reaction without hindrance, a cavity *x*, located above *A* was moved away from the reaction site (see label-2 in Fig. 2a). Further, cavity *C* was separated from dimer *C*–*y* by carefully pushing it away with the cantilever (see label-3 in Fig. 2a). To construct the *B*–*C* dimer, at first, the cantilever was positioned in such a way as to push cavity *A* away from *A*–*B* with a gentle force (Fig. 2 d). Fig. 2e shows the isolated *B* cavity from *A*, wherein the location of cavity *A* is below the cantilever. Then, cavity *C* (shown in Fig. 2d) was moved close to *B* in a step-wise manner to make a *B*–*C* dimer (Fig. 2f–h and j). Excitation of cavity *C* of *B*–*C* at the extreme left edge with a 405 nm laser produced bright PL in *C* and weak PL in cavity *B* (Fig. 4d). The PL spectra with WGMs were collected at positions *i*–*vi* (Fig. 4g and h). Though in the line spectra (Fig. 4g) the wavelengths of each peak at positions *ii* and *iii* appear the same and resemble those in spectrum *B*, the TE and TM mode intensities at position *iii* appeared nearly equal in contrast to the spectrum at position *ii* (Fig. 4h). The PL spectra at *v* and *vi* resemble the spectrum of cavity *C*, except for the TE/TM mode intensities. However, at position *i*, a mixed spectrum close to the spectra at positions *v*/*vi* and *ii*/*iii* was obtained. Interestingly, at the coupling point *iv*, between *B* and *C*, a nearly TM-polarized emission was observed. Notably, at position *i*, the modes with the azimuthal numbers 21 and 31 corresponding to *C* and *B* appeared split (the blue line shows the maxima of peaks with TM21 and TM31, respectively). FDTD calculations suggested the possible mode splitting by presenting bonding and anti-bonding of the modes from *C* and *B* with azimuthal numbers 21 and 31, respectively (Fig. S1†).

**Fig. 5:**
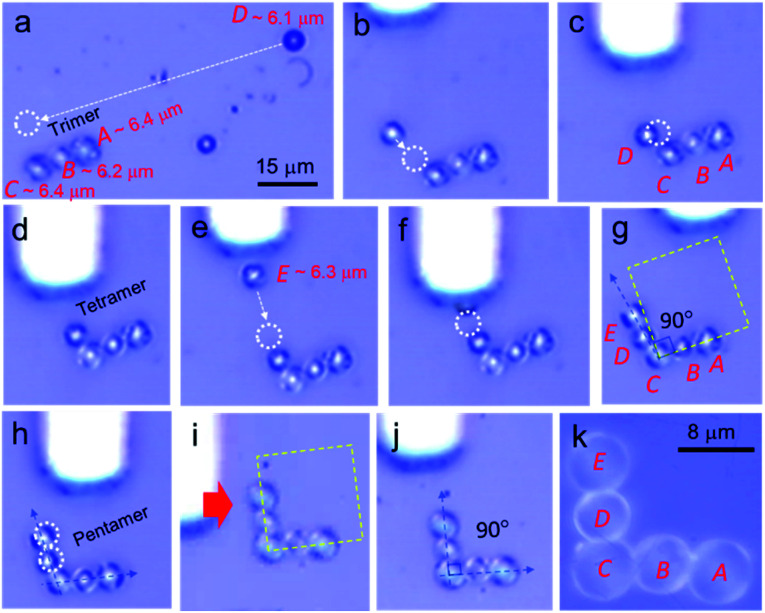
(a–c) Micromanipulation of cavity *D* towards a WGM cavity trimer, *A*–*B*–*C*, with an AFM cantilever; (c) positioning of cavity *D* adjacent to *C* at a ∼100° angle forming tetrameric cavities. (d) Adjustment of the position of *D* at a 90° angle to the *A*–*B*–*C* normal; (e and f) positioning of cavity *E* adjacent to *D* to form an L-shaped CROW. (g) L-shaped CROW comprising pentameric cavities with a ∠DCB angle of 101°. (h and i) Adjustment of the position of *D* and *E* to make a ∠DCB angle of 90°. (j) L-shaped CROW with a ∠DCB angle of 90°; (k) a close-up view of the L-shaped CROW made from five WGM cavities *A* to *E*.

Transmission of light through an L-shaped optical waveguide, wherein the two out-coupling terminals are oriented at a right angle (90°) to each other often leads to a substantial bending-induced optical loss.^2*d*^ An L-shaped CROW with five different optical cavities (pentamers) is an efficient and alternative approach to transmit the optical signal through a 90° bend angle. However, fabrication of complex CROW structures remains a challenging exercise, which includes techniques like lithography. Therefore, we extended the mechanical micromanipulation technique to assemble individual microcavities into an L-shaped CROW geometry. We identified a self-assembled WGM-cavity trimer of nearly comparable sizes, *A*–*B*–*C* with diameters of 6.4, 6.2, and 6.4 μm, respectively. Another cavity, namely *D* with a diameter of 6.1 μm, whose size is comparable with those of trimeric cavities, was identified and moved close (about a 10 μm distance) to *C* of the trimer (Fig. 5a). Later, in a step-wise-manner, cavity *D* was pushed towards *C* of the trimer to place it orthogonal to the *A*–*B*–*C* axis, forming tetrameric cavities (Fig. 5b–d). Similarly, cavity *E* with a size of 6.3 μm also was moved close to *D* and placed in such a way that it forms a nearly L-shaped structure comprising pentameric cavities (Fig. 5e–g). To create a perfect 90° angle between *A*–*B*–*C* and *C*–*D*–*E*, the positions of cavities *D* and *E* were slightly adjusted to complete L-shaped CROW geometry (Fig. 5i–k).

**Fig. 6:**
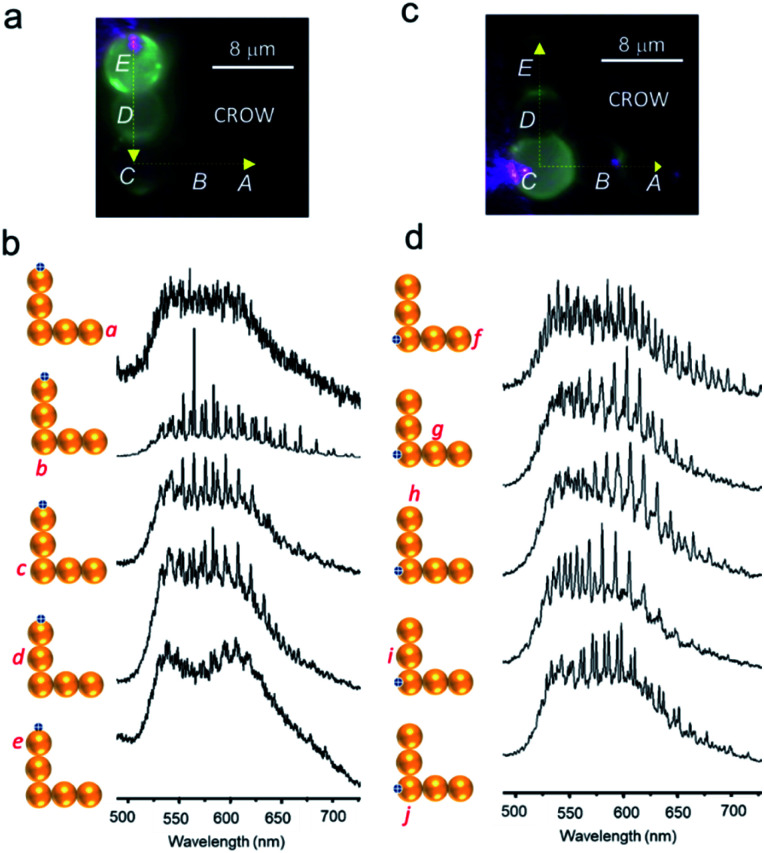
(a and c) PL images of L-shaped CROWs comprising pentameric cavities (*A*–*E*) optically excited at the periphery of cavities *E* and *C*, respectively. (b and d) PL spectra at different locations (*a*–*j*) of the CROW.

Optical excitation of cavity *E* of the CROW produced vivid PL and a WGM spectrum (see label *e* in Fig. 6a and b). The adjacent cavity *D* also displayed PL *via* evanescent field coupling and the PL spectra recorded at position *d* also sustained its WGMs. The corner cavity *C* of the CROW exhibited PL spectra at positions *c* and *b* with WGMs (Fig. 6b). Importantly, about 16% of the input light was transmitted to cavity *A* with weak WGM signatures (see position *a* in Fig. 6b). It should be mentioned that the light transmission efficiency could be higher if the light is coupled to cavity *E via* a tapered optical fibre. Here, the CROW can also be used to split and transduce optical signals into two termini (*f* and *h* in Fig. 6b). Optical excitation of the CROW at the corner cavity *C* showed split signals at output termini with nearly the same intensity. Further, cavity *C* exhibited both TE/TM modes at excitation position *j* and TM polarized signals covering ∼575–650 nm at positions *i* and *g*. Interestingly, at the out-coupling points *f* and *h*, a different set of WGM signatures were obtained wherein the signals in the ∼575–650 nm range were TM polarized for cavity *E* but unpolarised for cavity *A*.

## Conclusions

We introduced an *in situ* technique to mechanically micromanipulate dye-doped polymer WGM cavities of a size as low as 3 μm *via* a tip-less AFM cantilever and investigated the cavities' photonic properties by carrying out single-particle spectroscopy studies. Our method gives access to mechanically assemble or disassemble microcavities of our choice as a dimer, trimer, tetramer and pentamer and also in linear and bent geometries. For the first time, we were able to demonstrate the possibility of performing photon molecular reactions (PMRs) and constructing an L-shaped coupled-resonator optical waveguide (CROW) for spectral engineering and transduction of light at extreme bending, respectively. The presented technique can be applied to micromanipulate microcavities, lasers or waveguides of different sizes, shapes and chemical compositions to fabricate various CROWs, photonic circuits, microlaser arrays, optical filters, and multiple sensors. Finally, this mechanical manipulation technique can also be extended to any type (hard or soft) of microparticle relevant to various research areas.

## Conflicts of interest

There are no conflicts to declare.

## Supplementary Material

NA-002-D0NA00560F-s001
