# Supplementary material for: Mechanophotonics: precise selection, assembly and disassembly of polymer optical microcavities via mechanical manipulation for spectral engineering
Source: Nanoscale Adv. 2020 Oct 14;2(12):5584–90. doi: 10.1039/d0na00560f (PMC9417610; doi:10.1039/d0na00560f)
Supplement: NA-002-D0NA00560F-s001 [file NA-002-D0NA00560F-s001.pdf]

Electronic Supplementary Material (ESI) for Nanoscale Advances.
This journal is © The Royal Society of Chemistry 2020

Supporting Information

Mechanophotonics: Precise-Selection, Assembly and Disassembly of Polymer Optical Microcavities via Mechanical Micromanipulation for Spectral Engineering

*M. Annadhasan, A. Vinod Kumar, D. Venkatakrishnarao, E. A. Mamonov, R. Chandrasekar**

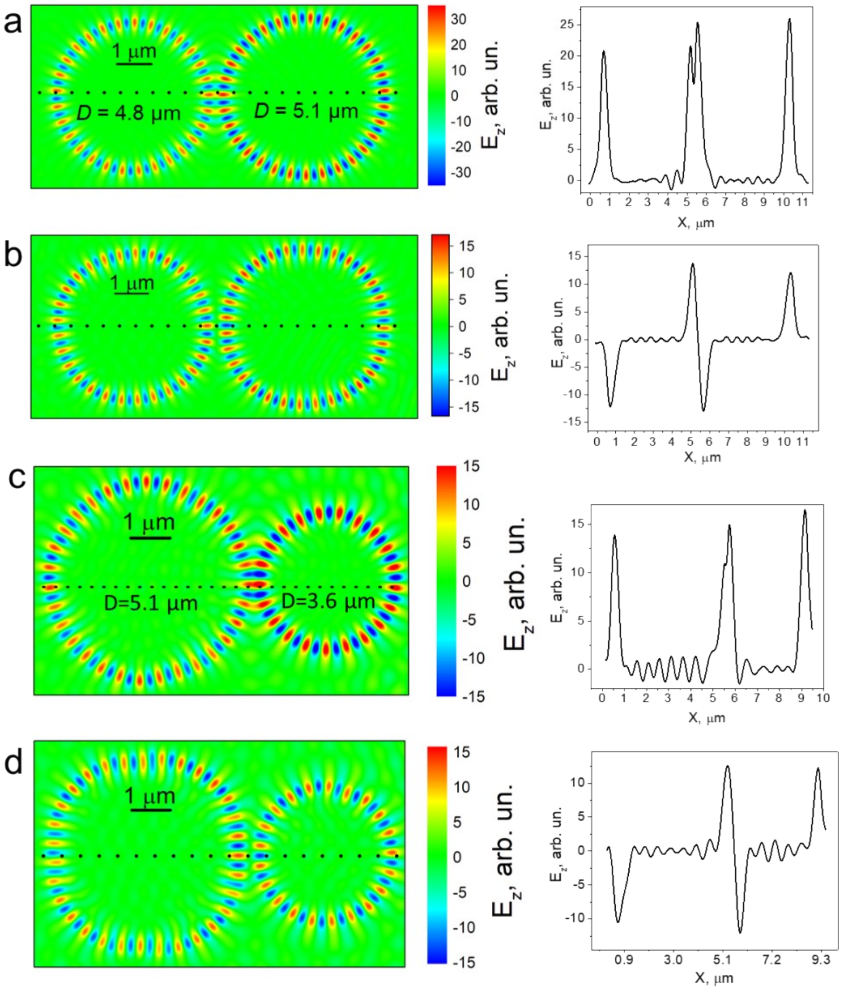

Figure S1. a and b) Calculated electric field (E_z) distributions for bonding and anti-bonding modes in the dimer for TM-31 and TM-29 modes corresponding to cavities *A* and *B*, respectively. c and d) Calculated electric field (E_z) distributions for bonding and anti-bonding modes in the dimer for TM-31 and TM-21 modes corresponding to cavities *B* and *C*, respectively. The considered distance between the spheres is 50 nm.

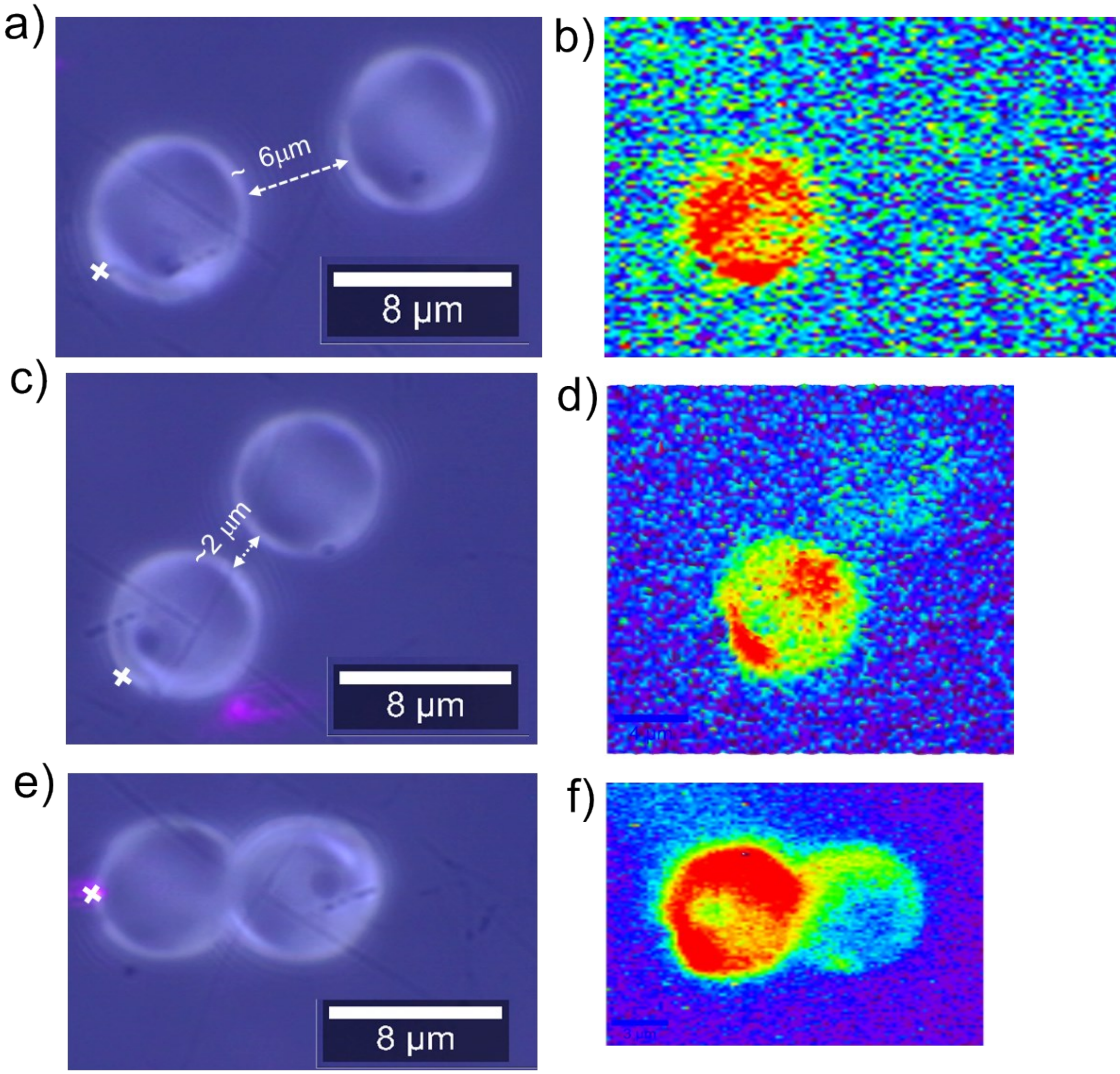

Figure S2. a, c, and e) Adjusting the gap between the WGM cavities via AFM cantilever. The “x” label indicates the excitation position. The corresponding FL images are shown in b, d, and f).
